# Relatively high prevalence of subclinical iron deficiency in Japanese children: evidence from a preoperative screening cohort

**DOI:** 10.1186/s12887-026-07080-2

**Published:** 2026-06-01

**Authors:** Yamato Hanawa, Yuji Baba, Wataru Murasaki, Hiroyuki Namba, Kimihiko Oishi

**Affiliations:** 1 Department of Pediatrics, Jikei University Kashiwa Hospital, Chiba, Japan; 2 Department of Surgery, Jikei University Kashiwa Hospital, Chiba, Japan; 3 Department of Pediatrics, The Jikei University School of Medicine, Tokyo, Japan

**Keywords:** Iron deficiency, Pediatric surgery, Risk assessment, Neurodevelopment

## Abstract

**Background:**

Iron deficiency (ID), even in the absence of anemia, is one of the most common micronutrient deficiencies in childhood and remains particularly relevant during infancy and early childhood, when brain development is rapid. Iron plays an essential role in myelination, neurotransmitter synthesis, mitochondrial function, and neuronal energy metabolism. Contemporary longitudinal and mechanistic studies suggest that early-life iron deficiency may be associated with later deficits in attention, executive function, and educational performance, although the magnitude and reversibility of these effects remain incompletely defined.

**Methods:**

We retrospectively reviewed pediatric patients who underwent elective surgery between January 2021 and June 2024. Patients with hematologic disorders, severe chronic disease, ongoing iron therapy, clinically active inflammatory conditions, or preoperative C-reactive protein (CRP) > 1.0 mg/dL were excluded. ID was defined as age-specific low serum ferritin and/or transferrin saturation (TSAT) < 16%. Anemia was defined using the 2024 World Health Organization hemoglobin cutoffs. Cases with discordant ferritin and TSAT results were classified as ID if either criterion was abnormal.

**Results:**

Of 199 eligible patients, 129 were included (76 males, 53 females). The majority were aged 6 months to 4 years. ID was identified in 26 children (20.2%), including 23 with ID without anemia and 3 with IDA (2.3%); all were asymptomatic. The ID group showed lower hemoglobin, MCV, MCH, serum iron, and ferritin, and higher RDW-CV, platelet count, and UIBC than the non-ID group.

**Conclusions:**

Subclinical ID was common among asymptomatic Japanese children undergoing elective surgery, indicating that clinically silent iron deficiency may be present even in children who appear otherwise well. Rather than supporting surgery-based screening as a stand-alone strategy, these findings underscore the importance of careful dietary and clinical risk assessment during routine pediatric care and the need for context-sensitive approaches to early identification of children at risk for iron deficiency.

## Background

Iron deficiency (ID), even in the absence of anemia, is among the most common micronutrient deficiencies worldwide in infants and young children [1]. It is well established that iron plays a critical role in early neurodevelopment, particularly in processes such as myelination, neurotransmitter function, and energy metabolism in the brain [2–3]. Recent studies have also suggested that iron deficiency without anemia may negatively affect motor development and behavior in infants and toddlers, underscoring the clinical relevance of subclinical ID [4–6].

Although many countries, including the United States, have recommendations for early childhood screening for anemia/IDA [7], some countries, including Japan, do not have a universal screening policy for iron deficiency. Even during routine pediatric visits, iron status is not routinely evaluated unless anemia is suspected or other clinical indications are present in Japan. Consequently, many asymptomatic children with ID may remain undiagnosed, potentially missing a critical window for early intervention.

Assessment of pediatric iron status requires careful interpretation because commonly used biomarkers reflect different aspects of iron metabolism and have important limitations. Serum ferritin is widely used as an indicator of iron stores, but it is also an acute-phase reactant and may be elevated in the presence of inflammation. Transferrin saturation (TSAT) may complement ferritin by reflecting circulating iron availability and iron-restricted erythropoiesis; however, TSAT can be influenced by diurnal variation, recent intake, and changes in total iron-binding capacity (TIBC). In particular, reduced hepatic protein synthesis in protein-energy malnutrition may affect TIBC and thereby influence TSAT. These considerations are important when defining ID in apparently healthy children [8–9].

To date, there is a lack of epidemiological data on the prevalence of ID with/without anemia in Japanese children without underlying conditions. Most previous studies in Japan have focused primarily on overt anemia, with limited attention given to the early stage of iron depletion. Understanding the actual burden of ID among otherwise healthy children is essential to guide screening strategies and to prevent ID-related developmental delays.

Obtaining reliable data on anemia and/or iron status in young children is challenging, as it typically requires invasive blood sampling, which is difficult to justify in the absence of clinical symptoms. However, preoperative blood testing for elective surgical procedures provides a valuable opportunity to assess hematological parameters and iron status in children without known medical conditions. This study aimed to determine the prevalence of ID in asymptomatic Japanese children undergoing elective surgery and to characterize their hematological profiles using routine laboratory screening.

## Methods

### Study design and population

This retrospective cross-sectional study was conducted in accordance with the Declaration of Helsinki. It was approved by the Ethics Committee of the Jikei University School of Medicine for Biomedical Research (#36–344). This study was conducted at Jikei University Kashiwa Hospital, a university-affiliated tertiary referral hospital in Chiba, Japan, where common pediatric elective surgical conditions are evaluated in a preoperative setting. We included pediatric patients who underwent elective surgery there between January 2021 and June 2024. Eligible participants were children aged 6 months to 14 years who underwent preoperative screening that included Hb, ferritin, serum iron, TIBC, unsaturated iron binding capacity (UIBC), and red blood cell distribution width (RDW), and who had no clinical symptoms of anemia (asymptomatic). We excluded children with (1) known hematologic disorders; (2) chronic conditions, such as chronic kidney disease or liver disease; (3) current use of oral iron supplementation; (4) use of anti-inflammatory medications; (5) antibiotic administration before preoperative blood testing; (6) clinically active inflammatory conditions such as appendicitis; (7) C-reactive protein (CRP) level > 1.0 mg/dL on preoperative testing; (8) incomplete clinical or laboratory data. These exclusions were applied to minimize potential distortion of iron indices by acute infection or inflammation and to improve the interpretability of ferritin- and TSAT-based definitions.

### Data collection

Demographic and clinical data, including age, sex, type of surgery, and laboratory parameters, were collected from the medical records. Laboratory tests included white blood cell (WBC), red blood cell (RBC) count, Hb, hematocrit (Ht), Mean Corpuscular Volume (MCV), Mean Corpuscular hemoglobin (MCH), Mean Corpuscular hemoglobin concentration (MCHC), platelet (PLT) count, platelet distribution width (PDW), RDW, Fe, TIBC, UIBC, serum ferritin, and CRP.

CRP values were subject to a lower detection limit in our laboratory assay and were therefore summarized descriptively. Because of this floor effect, CRP was not emphasized in the final between-group interpretation. Serum albumin levels were also measured as part of the routine preoperative laboratory assessment and were reviewed as a general indicator of nutrition status.

### Definitions

In our institution, serum iron, TSAT, and ferritin were measured as part of routine preoperative laboratory screening. Anemia was defined according to the World Health Organization (WHO) age-specific hemoglobin thresholds: Hb < 10.5 g/dL for children aged 6 months to 23 months, Hb < 11.0 g/dL for children aged 24 months to 59 months, Hb < 11.5 g/dL for children aged 5–11 years, or Hb < 12.0 g/dL for children aged 12–14 years [9].

Iron deficiency (ID) was defined as serum ferritin < 12 ng/mL (< 5 years), < 15 ng/mL (≥ 5 years), or TSAT < 16% [1, 10]. Iron deficiency anemia (IDA) was defined as anemia with concurrent ID. When ferritin and TSAT were discordant, no single biomarker was prioritized; children were classified as having ID if either criterion was abnormal.

CRP was used to improve interpretation of ferritin values in this retrospective dataset. Because ferritin is an acute-phase reactant and inflammation-adjusted ferritin thresholds or alpha-1-acid glycoprotein measurements were not available for all patients, children with CRP > 1.0 mg/dL were excluded to reduce misclassification due to concurrent inflammation.

### Group classification

After applying the inclusion and exclusion criteria, patients were classified into the ID or non-ID group according to the criteria described above.

### Statistical analysis

The Shapiro–Wilk test was used to assess the normality of continuous variables. Normally distributed data were presented as mean (SD) and compared between the two groups using unpaired two-tailed t-tests. Non-normally distributed data were presented as median (interquartile range) and compared using the Mann–Whitney U test. Categorical variables were expressed as numbers and percentages and compared using the Fisher’s exact test.

All *p*-values were two-sided, and *p* < 0.05 was considered statistically significant. All statistical analyses were performed using EZR (Saitama Medical Center, Jichi Medical University, Saitama, Japan) [11], which is a graphical user interface for R (The R Foundation for Statistical Computing, Vienna, Austria, version 4.3.1). EZR is a modified version of R Commander (version 1.6-8) designed to include statistical functions commonly used in biostatistics.

## Results

### Patient characteristics

A total of 199 pediatric patients were admitted for elective surgery between January 2021 and June 2024. After applying the exclusion criteria, 129 patients were included in the analysis (76 males, 53 females). The age distribution was as follows: 6 months to 23 months, 22.5%; 24 months to 59 months, 35.7%; 5 to 11 years, 40.3%; and 12 to 14 years, 1.6%. The most common surgical procedure was inguinal or umbilical hernia repair (81%), followed by undescended testis or hydrocele repair (16%), circumcision (2%), labial adhesion correction (1%), and surgery for cholelithiasis (1%) (Table 1). The median serum albumin level was 4.4 g/dL (IQR 4.3, 4.6), and no patient showed clinically significant hypoalbuminemia.

**Table 1:** Baseline demographic and surgical characteristics according to iron deficiency status. Data are presented as number of patients. *P* values were calculated using Fisher’s exact test for comparisons between the ID and non-ID groups

Characteristics	All (*n* = 129)	ID (*n* = 26)	Non-ID (*n* = 103)	*p* value
Sex				< 0.05
Male	76	20	56	
Female	53	6	47	
Age				0.09
6 months-23 months	29	12	17	
24 months-59 months	46	8	38	
5–11 years	52	6	46	
12–14 years	2	0	2	
Surgery type				0.83
Inguinal/umbilical hernia	105	21	84	
Undescended testis/hydrocele	20	5	15	
Circumcision	2	0	2	
Labial adhesion	1	0	1	
Cholelithiasis	1	0	1	

When stratified by iron deficiency status, sex distribution differed significantly between groups (*p* < 0.05), with males accounting for 76.9% (20/26) of the ID group and 54.4% (56/103) of the non-ID group. In contrast, age-group distribution (*p* = 0.09) and surgery type (*p* = 0.83) did not differ significantly between the two groups (Table 1).

### Prevalence of iron deficiency and iron deficiency anemia

Based on laboratory findings, 26 patients (20.2%) met the criteria for iron deficiency (ID), including 23 with ID without anemia and 3 with iron deficiency anemia (IDA, 2.3%). All children were asymptomatic at the time of preoperative evaluation. Among the 26 children classified as ID, 2 met the ferritin criterion alone, 22 met the TSAT criterion alone, and 2 met both criteria. Overall, low TSAT was observed in 24 children, whereas low ferritin was observed in 4 children.

### Comparison between ID and non-ID groups

Laboratory comparisons between patients in the ID group and those in the non-ID group are shown in Table 2. Consistent with Table 1, the proportion of males was significantly higher in the ID group than in the non-ID group (76.9% vs. 54.4%, *p* < 0.05). WBC, RDW-CV, PLT, and UIBC levels were significantly higher in the ID group (*p* < 0.01). In contrast, Hb, Ht, MCV, MCH, Fe, and ferritin levels were significantly lower in the ID group than in the non-ID group (*p* < 0.05). No significant differences were observed in RBC, MCHC, PDW, RDW-SD, or TIBC between the groups.

**Table 2:** Comparison of hematologic and iron-related parameters between children with and without iron deficiency. Continuous variables are presented as median (IQR) unless marked with an asterisk (*), in which case they are presented as mean (SD)

Variables	All (*n* = 129)	ID (*n* = 26)	Non-ID (*n* = 103)	*p* value
Male (*n*, %)	76, 58.9	20, 76.9	56, 54.4	< 0.05
WBC (×10^9^/L), median (IQR)	7.4 (6.2, 9.6)	9.3 (6.9, 12.7)	7.3 (6.0, 9.1)	0.002
RBC* (×10^12^/L), mean (SD)	4.8 (0.3)	4.8 (0.4)	4.7 (0.3)	0.09
Hb (g/dL), median (IQR)	12.8 (12.3, 13.3)	12.6 (12.1, 13.0)	12.9 (12.5, 13.4)	0.02
Ht (%), median (IQR)	37.8 (36.3, 39.1)	37.0 (35.8, 38.0)	38.2 (36.5, 39.4)	0.01
MCV (fL), median (IQR)	79.7 (77.5, 82.1)	76.9 (73.8, 78.6)	80.3 (78.4, 82.6)	< 0.001
MCH (pg), median (IQR)	27 (26.2, 27.7)	26 (25.0, 26.5)	27.2 (26.5, 28)	< 0.001
MCHC* (%), mean (SD)	33.9 (0.9)	33.8 (1.0)	33.9 (0.8)	0.5
PLT (×10^9^/L), median (IQR)	317 (274, 374)	388 (316, 418)	306 (263, 354)	< 0.001
PDW (%), median (IQR)	10.1 (9.4, 10.6)	9.9 (9.1, 10.4)	10.2 (9.4, 10.6)	0.2
RDW-CV (%), median (IQR)	13.3 (12.8, 13.9)	13.7 (13.3, 14.4)	13.1 (12.7, 13.8)	0.003
RDW-SD (fL), median (IQR)	38.7 (37.1, 40.2)	38.7 (36.8, 40.4)	38.7 (37.2, 40.1)	0.6
Fe* (µg/dL), mean (SD)	83.4 (32.7)	42.3 (12.1)	93.8 (27.6)	< 0.001
TIBC* (µg/dL), mean (SD)	335.5 (37.5)	347.0 (35.3)	332.5 (37.4)	0.08
UIBC* (µg/dL), mean (SD)	252.0 (49.79)	304.7 (38.1)	238.7 (43.1)	< 0.001
Ferritin (ng/mL), median (IQR)	26 (20, 35)	23 (15.8, 29)	27 (22, 38)	0.03

## Discussion

This study demonstrated that 20.2% of asymptomatic Japanese children undergoing elective surgery exhibited laboratory evidence of ID, whereas only 2.3% had progressed to overt IDA. The principal implication of this study is not that preoperative children constitute a special screening population, but rather that clinically silent ID may be present even among children who appear otherwise well and are receiving routine medical care.

An important point in interpreting our results is that most ID cases were identified on the basis of low TSAT rather than low ferritin. Specifically, 22 of the 26 children with ID met the TSAT criterion alone, whereas only 4 had low ferritin and only 2 met both criteria. This explains why the median ferritin value in the ID group remained above the ferritin cutoff. It also means that the estimated prevalence in this study should be interpreted as reflecting a broad biochemical definition of possible early iron deficiency rather than depleted iron stores alone. Because TSAT may be more sensitive to early functional iron restriction but is also more variable than ferritin, prevalence estimates may differ depending on the biomarker framework used [1, 8, 10].

The statistically significant laboratory differences observed between the ID and non-ID groups were modest in absolute magnitude. Their clinical significance lies less in any single abnormal value than in the overall pattern consistent with early iron depletion: lower Hb, Ht, MCV, MCH, serum iron, and ferritin, accompanied by higher RDW-CV and UIBC. In asymptomatic children, such findings are better interpreted as evidence of subclinical iron depletion at the group level rather than as individually decisive diagnostic markers.

Dietary factors likely contribute to pediatric iron deficiency in Japan; however, because our study did not include standardized dietary assessment, we could not determine which specific nutritional practices were involved [12]. In young children, insufficient intake of bioavailable iron during periods of rapid growth may be one plausible explanation. Lower intake of heme iron-rich foods and broader dietary patterns with limited iron bioavailability may increase vulnerability to early iron depletion. Because tea consumption was not assessed in this study and is likely to be less relevant in younger children, we avoided attributing the present findings to any single dietary habit [13]. Further studies incorporating detailed dietary assessment are needed to identify modifiable nutritional risk factors for subclinical iron deficiency in Japanese children [12].

The higher platelet count observed in the ID group may represent a nonspecific reactive finding that can accompany iron deficiency, rather than evidence of clinically important inflammation. Another finding that warrants cautious interpretation is the sex difference observed in this cohort. The proportion of males was significantly higher in the ID group than in the non-ID group. Previous studies have suggested that male infants may be more vulnerable to iron deficiency than females, possibly because of more rapid growth and higher iron requirements during early life. Domellöf et al. reported sex differences in iron status during infancy [14], Wieringa et al. found a higher prevalence of iron deficiency anemia among boys in infancy [15], and Guo et al. also reported sex-related differences in ferritin status in infants [16]. However, our finding should not be interpreted as evidence of a definitive biological sex effect in this cohort. The surgical case mix was male-skewed, particularly because inguinal or umbilical hernia and undescended testis/hydrocele accounted for a large proportion of procedures. Therefore, the observed sex difference may reflect both underlying biological susceptibility reported in infancy and selection related to the clinical composition of this preoperative cohort.

When compared with reports from other high-income settings, the prevalence observed in this cohort appears somewhat higher [17]. However, this comparison should be interpreted cautiously. Our cohort was not a population-based sample, but rather a single-center surgical cohort from a tertiary referral hospital. Therefore, the observed prevalence should not be interpreted as evidence that surgical patients have a surgery-specific biological risk of iron deficiency. Instead, the present cohort should be viewed as a practical clinical sample that allowed assessment of iron status in asymptomatic children who would not otherwise have undergone blood testing.

Iron plays an essential role in early neurodevelopment, including myelination, neurotransmitter synthesis, and cerebral energy metabolism [5, 18, 19]. Accumulating evidence indicates that ID, even in the absence of overt anemia, is associated with impaired cognitive and motor development [4–6, 20]. Importantly, recent clinical studies in toddlers suggest that timely iron supplementation can lead to measurable improvements in neurodevelopmental outcomes, indicating the importance of detecting ID before progression to IDA [6].

Despite the recognized developmental importance of iron status in early childhood, Japan does not currently have a standardized national screening program for pediatric iron deficiency. Our findings suggest that relying exclusively on clinically apparent anemia may miss children with earlier biochemical abnormalities. At the same time, routine universal screening for iron deficiency poses practical challenges because iron studies require blood sampling. In this context, the preoperative setting in this study should be viewed primarily as a practical observational window into iron status among otherwise asymptomatic children, rather than as a screening strategy in itself. Future work should focus on identifying feasible, low-burden risk-based approaches within existing child health systems and on determining which biomarker strategy is most appropriate for real-world pediatric practice [1, 7].

Several countries have issued national-level recommendations or conduct surveillance that facilitates early detection of ID/IDA. In the United States, the American Academy of Pediatrics recommends universal screening for IDA at around 12 months of age, reflecting the high vulnerability to ID in infancy and toddlerhood [7]. In Korea, analysis of nationally representative KNHANES data indicates that IDA is substantially more relevant in female aged ≥ 10 years than in males, underscoring the importance of age- and sex-specific risk stratification in population monitoring [21]. By contrast, Japan does not have a standardized national screening program for IDA. These differences in national approaches may influence detection rates and downstream management of pediatric iron deficiency [22].

This study has several limitations. First, because children with CRP > 1.0 mg/dL were excluded, our findings are not directly applicable to children with concurrent inflammation or infection. Second, this was a single-center study conducted in a tertiary referral hospital and may not fully represent the broader Japanese pediatric population. Third, although serum albumin was available as a general nutritional marker, formal anthropometric assessment and standardized dietary data were not systematically collected; therefore, subtle nutritional differences, including mild protein-energy malnutrition, could not be fully evaluated. Fourth, the prevalence estimate was driven predominantly by low TSAT rather than low ferritin, and different biomarker definitions may yield different estimates of iron deficiency burden. Finally, the cross-sectional design precluded assessment of long-term neurodevelopmental outcomes [3, 20].

Future multicenter, prospective studies incorporating detailed dietary assessments and longitudinal neurodevelopmental follow-up are warranted to confirm and extend our findings.

## Conclusions

This study demonstrates a substantial burden of subclinical iron deficiency among asymptomatic Japanese children undergoing elective surgery. The findings should not be interpreted as support for surgery-based screening as a primary strategy. Rather, they suggest that clinically silent iron deficiency may remain unrecognized in children who appear otherwise healthy, underscoring the importance of careful dietary and clinical risk assessment during routine pediatric care. Context-sensitive, risk-based approaches within existing child health systems may provide a more appropriate path toward earlier detection and intervention.

## Data Availability

The datasets generated and/or analyzed during the current study are not publicly available but are available from the corresponding author on reasonable request.

## Abbreviations

CRP: C-reactive protein
CV: Coefficient of variation
Hb: Hemoglobin
Ht: Hematocrit
ID: Iron deficiency
IDA: Iron deficiency anemia
MCH: Mean corpuscular hemoglobin
MCHC: Mean corpuscular hemoglobin concentration
MCV: Mean corpuscular volume
PDW: Platelet distribution width
PLT: Platelet
RBC: Red blood cell
RDW: Red blood cell distribution width
SD: Standard deviation
TIBC: Total iron binding capacity
TSAT: Transferrin saturation
UIBC: Unsaturated iron binding capacity
WBC: White blood cell
WHO: World Health Organization
